# Possible Beneficial Effects of N-Acetylcysteine for Treatment of Triple-Negative Breast Cancer

**DOI:** 10.3390/antiox10020169

**Published:** 2021-01-24

**Authors:** Youngjoo Kwon

**Affiliations:** Department of Food Science and Engineering, Ewha Womans University, Seoul 03760, Korea; Youngjoo.Kwon@ewha.ac.kr; Tel.: +82-2-3277-3103; Fax: +82-2-3277-4213

**Keywords:** N-acetylcysteine, triple-negative breast cancer, reactive oxygen species, antioxidant, tumor microenvironment

## Abstract

N-acetylcysteine (NAC) is a widely used antioxidant with therapeutic potential. However, the cancer-promoting effect of NAC observed in some preclinical studies has raised concerns regarding its clinical use. Reactive oxygen species (ROS) can mediate signaling that results in both cancer-promoting and cancer-suppressing effects. The beneficial effect of NAC may depend on whether the type of cancer relies on ROS signaling for its survival and metastasis. Triple-negative breast cancer (TNBC) has aggressive phenotypes and is currently treated with standard chemotherapy as the main systemic treatment option. Particularly, basal-like TNBC cells characterized by inactivated *BRCA1* and mutated *TP53* produce high ROS levels and rely on ROS signaling for their survival and malignant progression. In addition, the high ROS levels in TNBC cells can mediate the interplay between cancer cells and the tissue microenvironment (TME) to trigger the recruitment and conversion of stromal cells and induce hypoxic responses, thus leading to the creation of cancer-supportive TMEs and increased cancer aggressiveness. However, NAC treatment effectively reduces the ROS production and ROS-mediated signaling that contribute to cell survival, metastasis, and drug resistance in TNBC cells. Therefore, the inclusion of NAC in standard chemotherapy could probably provide additional benefits for TNBC patients.

## 1. Introduction

N-acetylcysteine (NAC) is widely used as a medication and a dietary supplement. It functions as a mucolytic due to its ability to break the disulfide bonds in the glycoproteins of the mucus, thus resulting in a decrease in viscosity [1]. In addition, NAC is used for the treatment of acetaminophen overdose because it can restore the depleted glutathione (GSH) reserves in the hepatocytes during the process of detoxification [2]. Moreover, NAC has been widely studied for its antioxidant effects [1,3,4]. This antioxidant activity is mainly attributed to its ability to act as a precursor of cysteine, which is itself a component of the primary intracellular antioxidant, GSH. Because the availability of cysteine can limit the rate of GSH synthesis under conditions of oxidative stress, the administration of NAC can replenish GSH stores [2,4]. By contrast, the direct antioxidant activity of NAC was thought to be irrelevant because the thiol group has inherently low reactivity toward oxidants [1]. However, an alternative mechanism has recently been suggested involving its ability to break the disulfide bonds of the cysteinylated proteins to release free thiols and regenerate reduced proteins, which can have direct antioxidant activity in certain cases (e.g., mercaptoalbumin) [1,5]. Another proposed mechanism involves the metabolic conversion of NAC-derived thiols into hyperactivated thiols (hydropersulfides) that act as direct oxidant scavengers and/or protective caps for critical protein thiols [6]. Therefore, NAC is a potent antioxidant due to both its direct antioxidant activity and the supply of a GHS precursor. Hence, NAC has been recommended for use in treating diseases where oxidative stress is involved at the onset and/or progression of the disease state [4]. The therapeutic use of NAC has been reported in several cancers, including breast cancer. For example, a pilot study has encouragingly suggested that NAC may be effective as a single agent in the inhibition of cancer cell proliferation in breast cancer patients [7].

Both the incidence of cancer and the number of cancer-related deaths are rapidly increasing worldwide, with breast cancer being the most frequently diagnosed. Approximately 2.1 million women were newly diagnosed with breast cancer in 2018, accounting for almost one in four cancer cases among women [8]. Due to early detection and effective treatment, the prognosis of breast cancer is relatively favorable. However, it is estimated that 627,000 women still died from breast cancer in 2018, which accounts for approximately 15% of all cancer-related deaths among women in that year [8]. Moreover, breast cancer is a highly heterogeneous disease in which certain types (e.g., triple-negative breast cancer) exhibit worse prognosis due to the lack of a targeted therapy [9]. Although NAC is well-tolerated and available at a low cost, the mechanisms underlying its efficacy in cancer treatment are complex and unclear. Moreover, NAC has been shown to exhibit cancer-promoting effects in some cancer types, including lung cancer [10] and melanoma [11], thus raising concerns regarding the clinical use of NAC. 

Antioxidants are generally regarded as cancer-preventive agents because they protect biomolecules from the detrimental effects of reactive oxygen species (ROS). However, antioxidants are also thought to protect cancer cells from ROS-induced cell death, thereby promoting their proliferation and malignancy. Nevertheless, this view may be an over-simplification of the functions of ROS [12,13]. ROS are known to trigger diverse responses that range from homeostasis to cell death. Therefore, it is important to define the types of cancers that rely on ROS for their survival and malignant behavior in order to facilitate the safe and efficacious use of NAC as a clinical treatment. Moreover, cancer also involves a complex interplay between the tumor microenvironment (TME) and the cancer cells. In this context, NAC may act on both the cancer cells and the TME. Hence, an understanding of the responses of both cancer cells and the TME to both ROS and NAC will be important for the therapeutic use of NAC. Hence, the present review briefly summarizes the functions of ROS as signaling molecules in order to discuss their roles in cancer development and progression. In addition, the current status of antioxidant use in cancer treatment is examined in order to highlight the importance of the selective use of NAC for specific types of cancer. Finally, the discussion focuses on those types of cancer that rely on ROS for cancer cell survival and the creation of a permissive environment that promotes the progression to malignant cancer. These are the types of cancer that can potentially benefit from the use of NAC. 

## 2. The Formation and Elimination of ROS 

Cellular ROS levels exist in a steady, dynamic equilibrium between their formation via diverse cellular processes and their elimination via the antioxidant system. As described in the following paragraphs, ROS are produced during a variety of cellular reactions and upon exposure to various extraneous agents including toxicants, drugs, and xenobiotics, or ultraviolet light and other forms of radiation [14]. All of these factors can influence the intracellular ROS levels. Moreover, complex interactions between multiple cellular sources of ROS can be involved in the regulation of ROS homeostasis. To appreciate the resulting dynamic changes in redox states, the various ROS species and their reaction products are briefly described, along with the major cellular sources of ROS formation. 

While the major ROS include the superoxide (O_2_^•−^), hydrogen peroxide (H_2_O_2_), and hydroxyl radicals (^•^OH) [15], other ROS include reactive nitrogen species (RNS) such as nitric oxide (NO^•^), peroxynitrite (ONOO^−^), and dinitrogen trioxide (N_2_O_3_). Among these, NO^•^ is generated by the family of nitric oxide synthetases [16]. Subsequently, NO⋅ can undergo autooxidation to form N_2_O_3_ and react with O_2_^•−^ to form ONOOˉ [17]. Individual ROS have distinct characteristics regarding their half-lives, localization, inherent chemical properties that confer reactivity to various biological targets, and elimination by antioxidant enzymes [18]. Although NO^•^ is relatively inert, its derivative, ONOO^−^ is a powerful oxidant that can damage many biological molecules [19]. The O_2_^•−^ is also highly reactive but short-lived. The O_2_^•−^ generated within cells is rapidly converted into H_2_O_2_ by local superoxide dismutases (SODs) [20,21]. By comparison, H_2_O_2_ is more stable than O_2_^•−^ but can be reduced to water in a reaction catalyzed by catalase (CAT) or glutathione peroxidase (GPX) [22]. The reaction involving GPX requires the oxidation of GSH to glutathione disulfide, which is subsequently reduced back to GSH by glutathione reductase, thioredoxin, and glutaredoxin [23,24]. Thus, the cell contains an enzyme system for preventing the build-up of H_2_O_2_ and maintaining a steady-state concentration (1–10 nM) [25]. 

Nevertheless, the presence of trace metals can catalyze the formation of the extremely reactive ^•^OH from H_2_O_2_ via the Fenton reaction. This ^•^OH cannot be eliminated enzymatically and can lead to cellular damage or genomic instability due to the indiscriminate oxidation of nearby lipids, proteins, and DNA [18,26]. In particular, ROS such as ^•^OH and ONOOˉ can oxidize nucleic acid bases or the deoxyribose backbone to potentially form oxidation products, strand breaks, and DNA-protein crosslinks [27]. The ONOOˉ reacts primarily with deoxyguanosine to cause modifications such as 8-oxo-2’-deoxyguanosine and 8-nitro-2’-deoxyguanosine [17]. In addition, ROS can modify protein structure and function via the nitration of tyrosine residues and the oxidation of critical cysteine residues [28]. Further, ROS can initiate lipid peroxidation via the formation of the fatty acid radical, which is highly unstable and readily reacts with molecular oxygen to produce the lipid peroxyl radical (LOO^•^). This unstable radical species reacts in turn with another free fatty acid to produce a different fatty acid radical and a lipid peroxide (LOOH) [29]. Thus, a free radical chain reaction generates lipid peroxides that can be decomposed to form reactive lipid electrophiles such as 4-hydroxy-2-nonenal (HNE). These breakdown products (e.g., HNE) can form covalent adducts with nucleophilic functional groups in proteins, nucleic acids and membrane lipids [29,30]. Moreover, their prolonged half-lives may allow them to serve as second-messengers of oxidative stress [31]. Therefore, ROS can react with cellular macromolecules and lead to biological and pathological effects. However, it should be also noted that the functional significance of many ROS reaction products in vivo still needs to be validated and is under active study [17]. 

ROS are generated in multiple cellular organelles including mitochondria, peroxisomes, the endoplasmic reticulum (ER), and the plasma membrane. A major source of ROS is the mitochondrial respiratory chain, a normal metabolic process that converts the energy stored in macronutrients into adenosine triphosphate [26]. During mitochondrial respiration, electrons are transferred from electron donors to electron acceptors via oxidation-reduction reactions. The last electron acceptor in this chain is an oxygen molecule, which is normally reduced to water. However, a small fraction of the consumed molecular oxygen undergoes incomplete reduction, leading to O_2_^•−^ formation [15]. 

Peroxisomes play a central role in various metabolic pathways including fatty acid oxidation, anaplerotic reactions, ether phospholipid synthesis, and hydrogen peroxide metabolism [32]. Many enzymes involved in these metabolic pathways produce specific ROS or RNS during their normal catalysis [33]. Peroxisomes also contain catalase and other antioxidant enzymes that effectively eliminate ROS [33]. Therefore, peroxisomes have the ability to rapidly produce and decompose ROS, thus allowing dynamic changes in ROS levels. Furthermore, peroxisomes and the mitochondria are metabolically interconnected and intimately related in redox regulation [34,35]. 

ROS are also produced in the ER during the metabolism of xenobiotics, the synthesis of unsaturated fatty acids, and protein folding [36]. A major source of ROS in the ER is oxidative protein folding that involves intramolecular and intermolecular disulfide bond formation [37]. For example, protein disulfide isomerase (PDI) is an ER oxidoreductase that catalyzes oxidative folding of protein. During disulfide bond formation, cysteine residues in the active sites of PDI accept two electrons from the cysteine thiol groups of nascent polypeptide substrates, leading to the reduction of PDI and oxidation of the substrate [38]. Subsequently, PDI can be regenerated in its oxidized form by the transfer of electrons to ER oxidoreductin 1 (Ero1), a flavin adenine dinucleotide (FAD)-binding protein [37]. After accepting electrons from PDI, Ero1 is re-oxidized by the transfer of electrons to molecular oxygen, but incomplete reduction results in the production of O_2_^•−^, which can be converted to H_2_O_2_ or other ROS [39,40]. 

In addition, NADPH oxidases (NOXs), a group of transmembrane proteins found in the plasma membrane, are recognized as major sources of cellular ROS [41]. Seven types of NOX are found in humans [42]. ROS production by NOXs is well recognized as a host defense function [43]. In addition to phagocytic cells, NOXs are expressed in non-phagocytic cells in a wide variety of tissues, albeit at much lower levels [44]. However, NOX activity becomes rapidly activated upon their assembly in membranes in response to various ligands such as cytokines, hormones, and growth factors [24,42]. The NOXs transfer electrons sequentially from cytosolic NADPH to FAD, then to each of two hemes, and finally to molecular oxygen on the opposite side of the cell membrane, thus producing O_2_^•−^ (NOX1–3 and NOX5) or H_2_O_2_ (NOX4 and dual oxidase 1–2) [41,42]. Several NOXs (e.g., NOX4) are also located in the ER membrane, where they catalyze the generation of ROS [45]. Therefore, a variety of ROS are produced from multiple cellular sources which cumulatively contribute to the physiological or pathological effects of ROS. 

## 3. The Function of ROS as Signaling Molecules 

In addition to pathophysiological responses, ROS-mediated signal transduction plays a role in various basal and adaptive physiological responses for organismal homeostasis [14]. Compared to other ROS molecules, H_2_O_2_ is relatively stable and has both reducing and oxidizing properties, making it a central redox signaling molecule [46]. Protein modification by H_2_O_2_ yields instrumental signaling intermediates and is specific to certain target proteins [20]. The best-studied mechanism by which H_2_O_2_ achieves specificity in signaling mediation occurs via the oxidation of cysteine (Cys) residues on target proteins [20,47]. At physiological pH, Cys residues exist in the form of thiolate anions (Cys-S^−^), which are more susceptible to oxidation than the protonated cysteine thiol (Cys-SH) [46]. The thiolate anion can be oxidized to the sulfenic form (Cys-SOH) by H_2_O_2_, leading to functional changes in target proteins. The sulfenic form can be reduced back to the thiolate anion by disulfide reductases such as thioredoxin and glutaredoxin to restore function [48]. Hence, a steady-state physiological flux of H_2_O_2_ (in the nM range) toward specific target proteins leads to reversible oxidation, thereby serving as a reversible signal transduction mechanism that results in the alteration of protein activity, localization, and interaction with other biomolecules [47]. However, persistence of the H_2_O_2_-mediated sulfenic form can lead to further oxidation to sulfinic (SO_2_H) or sulfonic (SO_3_H) forms. Unlike the sulfenic modifications, the formation of sulfinic and sulfonic species can be irreversible, thus leading to permanent protein damage [49]. The O_2_^•−^ has also been found to interact directly with specific intracellular targets and trigger signal transduction through the inactivation of specific proteins that are sensitive to O_2_^•−^ levels although it has poor reactivity due to a short half-life [50]. 

This ROS-mediated signaling can be physically and functionally compartmentalized within the cell [51]. For example, NOXs localize in specific subcellular compartments [42]. Their ROS production is activated by various ligands, and the resulting ROS serve to modulate proximate redox-sensitive targets in intracellular signaling pathways. In addition, antioxidant enzymes are localized in specific cellular locations. Both ROS production and elimination by antioxidant enzymes occur at specific sites in cells and are associated with specific stimuli. This spatial and temporal organization of ROS production and removal can determine the outcomes of ROS signaling [51]. However, ROS are also believed to rapidly diffuse across membranes via certain aquaporins (AQPs) or other specific channels [52,53]. Therefore, ROS generated in one compartment can trigger responses in another [54]. For example, ROS produced by mitochondria facilitate disulfide formation in cell surface proteins, which may regulate protein function [55]. In addition, peroxisome-derived H_2_O_2_ could oxidize redox-sensitive cysteine residues in multiple proteins including the nuclear factor kappa-light-chain-enhancer of activated B cells (NF-kB) and phosphatase and tensin homolog (PTEN) within as well as outside peroxisomes [56]. The production of peroxisomal ROS could also trigger mitochondrial apoptosis pathways [35]. Thus, ROS readily cross peroxisomal and mitochondrial membranes and can mediate signaling both within and outside the compartments in which they originate. 

In addition, ROS produced in one cell can affect neighboring cells. For instance, H_2_O_2_ diffuses through the AQP family, in a similar fashion to water [53]. By diffusing through these channels, H_2_O_2_ acts as a signaling molecule not only in an autocrine, but also in a paracrine fashion. Notably, H_2_O_2_ produced in myofibroblasts induces cell death in adjacent lung epithelial cells [57]. Upon co-culture with fibroblasts derived from psoriatic plaque lesions, the intracellular accumulation of ROS and the activation of ERK have been shown to result in the proliferation of keratinocyte [58]. The same study further revealed that ROS over-produced by NOX4 of the fibroblasts in psoriatic plaques act as mitogens for keratinocytes. Thus, intercellular signaling has been demonstrated between H_2_O_2_ donor and recipient cells. The O_2_^•−^ has a short lifetime due to rapid conversion to H_2_O_2_ and cannot diffuse across biological membranes due to its negative charge. Nevertheless, O_2_^•−^ has been shown to initiate intracellular signaling by its passage through anion channels in the cell membrane [59]. Thus, ROS generated in one cell can stimulate redox signaling in adjacent cells. 

## 4. The Effect of Antioxidant Supplementation upon Cancer Development and Progression

Excessive ROS can react with biomolecules including DNA, which is considered to be a possible cause for the increase in oncogenic mutations and the development of cancer [60]. This notion led to the expectation that dietary antioxidants might effectively prevent cancer development and has been a theoretical basis and explanation for the cancer-preventive effects of various antioxidant-rich plant foods observed in epidemiological studies. However, cancer-promoting effects have also been observed for antioxidants in some preclinical studies [10,11]. For example, supplementation with NAC or vitamin E after the development of cancer was found to increase cancer progression and reduce survival in mouse models of both *BRAF*- and *KRAS*-induced lung cancer [10]. Similarly, the oral administration of NAC after small nevi formation was shown to increase lymph node metastases without affecting the number and size of the primary tumors in a transgenic mouse model of melanoma [11]. These cancer-promoting effects of dietary antioxidants have resulted in a change in the perspective on the role of antioxidants in cancer. While antioxidants do prevent ROS-mediated cellular damage, they also protect cancer cells from ROS-mediated death. Therefore, antioxidants can effectively prevent cancer initiation, but can promote the growth of established cancers. 

Although the cancer preventive effect of dietary antioxidants has been demonstrated in some studies [61,62], their use has also been shown to be ineffective in decreasing the incidence of cancer [63,64]. Moreover, some antioxidants were found to increase the risk of cancer. For instance, a large phase III randomized placebo-controlled trial demonstrated that dietary supplementation with vitamin E for over 7 years significantly increased the risk of prostate cancer [65]. In addition, some preclinical studies have indicated that supplementation with antioxidants can increase the risk of cancer development [66,67]. Chronic NAC treatment was shown to increase cancer initiation both under abnormal conditions associated with lung oxidative stress (*JunD* deletion) and during normal aging in mice [66]. In this study, although NAC treatment decreased oxidative stress and cell senescence in the lungs, this led to increased cancer initiation. In addition, GSH synthesis driven by the glutamate cysteine ligase modifier subunit (GCLM) was shown to be necessary for cancer initiation in a transgenic *mammary tumor virus-polyoma middle tumor-antigen* (*MMTV-PyMT*) mouse model of breast cancer [67]. In contrast, genetic loss of *Gclm* significantly delayed the onset of mammary tumor and reduced the mammary tumor burden [67]. The same study further demonstrated that the mammary tumor burden was dramatically reduced by treatment with buthionine sulfoximine (BSO), a potent inhibitor of GSH synthesis, prior to the onset of cancer development, but not upon the onset of mammary tumor growth, thus suggesting the need for GSH in breast cancer development. These results mitigate against the use of antioxidants for the prevention of cancer and reveal an inconsistency in the anticipated beneficial effects of antioxidants in cancer initiation. 

Contradictory results have also been obtained with respect to the use of antioxidants in cancer progression. In a melanoma mouse model, cancer cells in metastatic sites (blood and viscera) had higher ROS levels than those in primary sites [68]. This high oxidative stress in metastatic cells was found to suppress distant metastasis, and treatment with an antioxidant (NAC, 200 mg/kg/day) increased the frequency of metastasis without significantly affecting the growth of established primary tumors [68]. However, in another study, the increased ROS levels in highly metastatic cells activated v-Src avian sarcoma (Schimidt-Ruppin A-2) viral oncogene homolog (Src), which led to increased cell migration and metastases. In contrast, mitochondrial superoxide scavenging by rotenone was shown to prevent ROS-mediated Src activation and consequently inhibit both the migration of cancer cells in vitro and spontaneous cancer metastasis in mouse xenograft models using melanoma and breast cancer cell lines [69]. Thus, the roles of ROS and antioxidants in cancer progression cannot be generalized. 

A recent study has clearly demonstrated a change in ROS effects depending on the stage of the cancer. Suppressing ROS production during high expression of the antioxidant TP53-inducible glycolysis and apoptosis regulator (TIGAR) promoted premalignant cancer initiation, but limited metastasis in a pancreatic ductal adenocarcinoma (PDAC) model [12]. In contrast, increased ROS production following the loss of TIGAR in PDAC cells drove a phenotype switch that increased invasive and metastatic capacity [12]. However, this might be related to a particular scenario where ROS production was under the control of TIGAR, whose expression changed during the progression of cancer. Overall, the role of ROS (or antioxidants) is inconsistent, both in cancer initiation and progression, and their effects cannot be generalized on the basis of the cancer stage. 

Cellular ROS can mediate cancer-promoting signaling and, thus, facilitate cancer cell proliferation and survival. For example, membrane-associated ROS generated via NOXs (e.g., NOX4) are important contributors to the activation of signaling pathways that drive proliferation and metastasis of cancer cells [70]. Mitochondrial ROS have also been shown to be necessary for *KRAS*-induced cancer development [71]. In contrast, ROS can induce cellular senescence and cell death, thereby acting as cancer-suppressing agents. Furthermore, cancer cells are believed to produce higher levels of ROS and hence, rely more on antioxidant activity to reduce their ROS burden than do non-cancerous cells [72]. Due to the potentially higher sensitivity of cancer cells to ROS-mediated cell death compared to normal cells, exogenous ROS generation has been proposed as a therapy for selectively killing cancer cells without affecting normal cells [73]. Therefore, ROS are considered to be a double-edged sword in cancer development and cancer treatment [74]. Consequently, treatment with antioxidants including NAC can be beneficial or detrimental in cancer management. 

## 5. The Therapeutic Potetial of NAC in TNBC 

Breast cancer is a heterogeneous disease [75,76], and this diversity is attributed to distinct genetic, epigenetic, and transcriptomic changes [77,78]. Molecular characteristics based on gene expression data led to the classification of the following molecular breast cancer subtypes: luminal A, luminal B, HER2 over-expression, and basal-like breast cancer [79,80]. These molecular subtypes are relatively well represented by their estrogen receptor (ER), progesterone receptor (PR), and human epidermal growth receptor 2 (HER2) statuses, which can be identified by immunohistochemistry. As such, luminal A is ER- and/or PR-positive and HER2-negative; luminal B is ER- and/or PR-positive and either HER2-positive or -negative; and HER2 over-expression or amplification is hormone receptor-negative and HER2-positive [78]. Basal-like cancers constitute an extremely heterogeneous group, and the majority (80%) of this type, which lack of ER, PR, and HER2 expression, are referred to as triple-negative breast cancers (TNBCs) [81,82].

The above breast cancer classification scheme provides guidance for patient treatment [83]. For example, luminal types depend on estrogen for their growth and can be treated with drugs to reduce either the estrogen action (e.g., tamoxifen) or the actual level of estrogen (e.g., letrozole) [83]. Breast cancer with HER2 amplification can be effectively treated with humanized anti-HER2 monoclonal antibodies or small molecule kinase inhibitors (e.g., trastuzumab) [84]. However, no molecular-based targeted therapy is available for TNBCs [9]. Thus, breast cancer is comprised of various subtypes associated with distinct molecular characteristics and treatment responses. Moreover, the content and reliance of cancer cells on ROS for their survival and progression may vary among the various cancer types. For some cancers, high levels of ROS may be necessary to support their growth and malignant behavior, whereas other cancers may be more effectively treated by induction of ROS-mediated death [72,85]. In addition, the development and progression of a cancer toward the malignant phenotype is known to be highly dependent on interactions with the TME [86,87,88,89]. The ROS can mediate TME-induced signal transduction to promote cancer progression [90]. High ROS levels derived from cancer cells may also result in the creation of a permissive TME by altering adjacent stromal cells to acquire cancer-associated phenotypes and by recruiting immune cells [91,92,93]. Therefore, breast cancers with high ROS production in either the cancer cells or the TME would benefit from a safe and efficacious NAC treatment that is tailored toward the specific cancer type. 

### 5.1. The Dependence on ROS for the Survival and Malignant Progression in TNBC

Otto Warburg was the first to describe a propensity for cancer cells to metabolize glucose anaerobically even in the presence of oxygen (the Warburg effect), and suggested an explanation for this abnormal glucose metabolism in terms of impaired bioenergetic activity of the mitochondria [94]. This led to the belief that oxidative phosphorylation (OXPHOS) is downregulated in cancer cells [95]. However, accumulating evidence suggests that aerobic glycolysis does not predict the loss of oxidative metabolism [96], and impairment of mitochondrial metabolism cannot be generalized across all cancers [97,98]. Moreover, increased OXPHOS and mitochondrial mass have been linked to the invasive and migratory potential of cancer cells [99]. 

**Figure 1:**
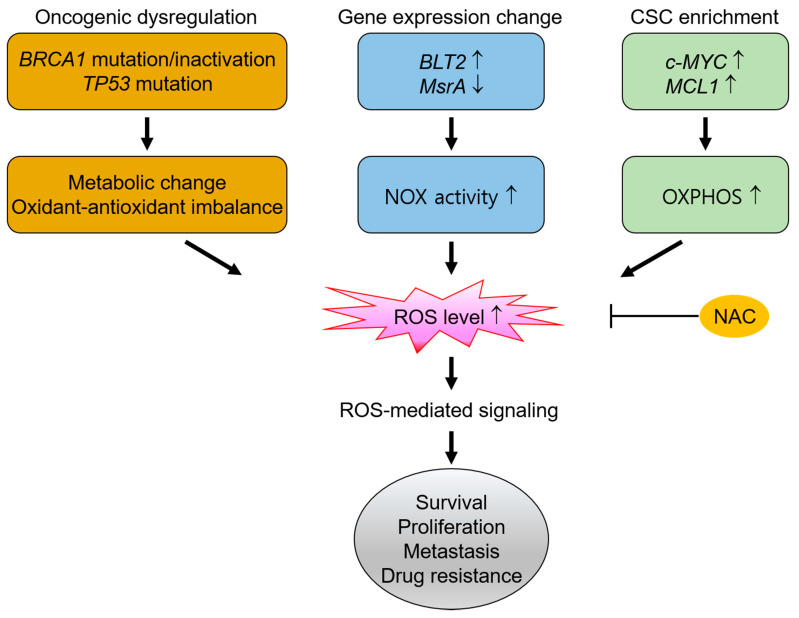
The dependence of triple-negative breast cancers (TNBCs) on reactive oxygen species (ROS) signaling for their survival and malignant progression. Basal-like TNBC is related to the *BRCA1 mutation*/inactivation and *TP53* mutation. This oncogenic dysregulation induces metabolic changes and oxidant-antioxidant imbalances that lead to high ROS production, which may be necessary for the survival and proliferation of TNBC cells. In addition, TNBC cells may further undergo gene expression changes (i.e., *MsrA* loss and *BLT2* amplification) that increase ROS production via NOX activity, which then modulates signaling that promote cell survival and invasion. High ROS levels also stabilize gene expression (e.g., *MMP-9*) related to the metastatic phenotype. Moreover, the high drug-resistant and metastatic properties of TNBC are often related to an increase in the cancer stem-like cell (CSC) fraction (i.e., overexpression of *c-MYC* and *MCL1*) that produces high amounts of ROS via high oxidative phosphorylation (OXPHOS). This ROS-mediated signaling leads to TNBC progression, but is effectively attenuated by N-acetylcysteine (NAC) treatment, thereby reducing the survival and metastasis of TNBC cells.

As noted above, TNBC comprises a very heterogeneous subgroup of cancers that accounts for 10–20% of all breast cancers [100,101]. Moreover, due to a lack of effective treatment options, TNBC is often associated with an aggressive phenotype and worse disease-specific outcomes than other breast cancer subtypes [102,103]. Significantly, one of the mechanisms that promote cell survival and drive aggressiveness in TNBC has been linked to high levels of ROS production resulting from gene mutations, gene expression changes and the attainment of stem cell-like properties [91,104,105]. This high ROS content induces multiple signaling which, in turn, leads to highly proliferative, migratory, and drug-resistant phenotypes in TNBC (Figure 1).

#### 5.1.1. High ROS Production Derived from BRCA1 Inactivation and TP53 Mutation in TNBC

High ROS production contributes to cancer cell survival and growth, particularly in basal-like TNBC [106]. Basal-like TNBC tumor samples from the Cancer Genome Atlas dataset exhibit distinctly different mitochondrial gene expression, thus suggesting differences in mitochondrial function in this subtype of breast cancer [106]. Basal-like TNBC cell lines (MDA-MB-231, MDA-MB-468, and BT-549) contain higher ROS levels than luminal (MCF-7) or non-cancerous (MCF-10A) cell lines, the majority of which are located in the mitochondria [106]. Treatment with NAC (15 or 30 mM) has been shown to induce cell death in TNBC cell lines but not in luminal cells, thus indicating the dependence of basal-like TNBC cells on high levels of ROS for their survival and proliferation [106]. In addition, increased ROS level have been found necessary for the migratory and invasive properties of MDA-MB-231 cells [99]. Elevated levels of mitochondrial DNA have been detected in circulating cancer cells from mice with tumors derived from orthotopically implanted MDA-MB-231 cells compared to primary tumors [99]. This high DNA content in metastatic cancer cells was attributed to high mitochondrial biogenesis and OXPHOS due to high expression of peroxisome proliferator-activated receptor gamma coactivator 1-α. The perturbation of mitochondrial respiratory function in breast cancer cells has also been shown to result in ROS generation [107]. This increased ROS was shown to promote breast cancer cell motility via a pathway mediated by activator protein-1 and C-X-C motif chemokine ligand (CXCL) 14, while pretreatment with NAC (5 μM) was shown to effectively inhibit this ROS-induced cell motility [107]. Thus, metabolic alteration induced by changes in mitochondrial function in basal-like TNBC cells accumulates high ROS levels that contribute to high proliferative and metastatic properties.

The inactivation of breast cancer susceptibility gene 1 (BRCA1) may also be related to high ROS content in basal-like TNBC. BRCA1 dysfunction is regarded as a drivers of basal-like breast cancer as well as a subgroup of TNBC [77,100,108]. Although *BRCA1* is infrequently mutated in sporadic TNBC, low expression or inactivation of BRCA1 may occur in wild-type TNBC due to epigenetic gene silencing, miRNA-mediated post-transcriptional suppression, and other nongenetic alterations [109,110,111]. In addition to its established function as a tumor suppressor, BRCA1 has been shown to regulate oxidative stress [112]. The overexpression of wild type, but not the cancer-associated mutant form, of BRCA1 was shown to significantly reduce the cellular levels of ROS and protein damage caused by H_2_O_2_, whereas the knockdown of *BRCA1* increased the ROS levels in MCF-7 and MCF-10A cells [113]. Thus, the protective role of BRCA1 against oxidative stress, in combination with the oxidative DNA-damaging ability of estrogen metabolites, may partially explain the specificity of *BRCA1*- and *BRCA2*-mutant cancers in the breast and ovary, where estrogen levels are relatively high [114]. This has been further demonstrated in a mouse mammary tumor model with specific knock-out of *Brca1* in the mammary gland [115]. The DNA double-strand breaks induced by oxidative estrogen metabolites could not be repaired without Brca1, thus resulting in genomic instability and cancer development. By contrast, antioxidant treatment (4-hydroxy-2,2,6,6-tetrametylpeperidine-N-oxyl, tempol) suppressed the levels of estrogen-induced oxidative DNA lesions, and significantly delayed the onset of Brca1-deficiency-induced cancer development [115]. Thus, increased ROS levels in basal-like subtypes of TNBCs may be associated with *BRCA1* mutation/inactivation and may be essential for the survival of this type of cancer.

In addition, TNBC exhibits high levels of genomic instability, with *TP53* being the most frequently mutated somatic gene (over 80%) [91,100,108]. The TP53 gene regulates cellular ROS levels by either controlling the expression of antioxidant and prooxidant genes or by modulating the metabolic pathways [116,117]. Cancer cells containing mutant *TP53* have been shown to exhibit increased mitochondrial biogenesis and OXPHOS, which resulted in increased metastasis [118]. Moreover, low p53 levels result in decreased transcription of antioxidant genes (e.g., *Mn-SOD* and *GPX1*), whereas high levels induce an imbalance of antioxidant gene transcription (i.e., upregulation of *Mn-SOD* and *GPX*, but not of *CAT*) and transactivation of prooxidant genes (e.g., *p53 upregulated modulator of apoptosis* and *p53-induced gene*). Thus, *TP53* mutation is associated with high cellular ROS levels due to an imbalance in the antioxidant defense [119,120]. Therefore, the *BRCA1* inactivation and *TP53* mutation that often observed in TNBC may contribute to a significant ROS content in these cells. Hence, the effective removal of ROS in TNBC by NAC treatment can suppress ROS signaling that leads to malignant progression of this type of cancer. Notably, *TP53* mutation is also prevalent in other types of cancer, including ovarian and esophageal cancer [121,122]. This suggests that survival and proliferation of these cancer cell types are ROS-dependent and that NAC treatment is of potential benefit in such cancers. 

#### 5.1.2. High ROS Production Derived from Gene Expression Changes in TNBC 

The enzymatic action of NOXs also leads to ROS production (Section 2), and gene expression changes that induce high NOX-related ROS generation have also been observed in TNBC [123,124]. These NOX-derived ROS have been shown to contribute to cell proliferation, survival, and invasive behavior in TNBC. For instance, the leukotriene B4 receptor 2 (BLT2) is highly expressed in breast cancer tissues at both the mRNA and protein levels [123]. Activation of BLT2 triggers signaling pathways that promote the survival, metastasis, and adhesion of cells, and its high expression is associated with a worsened prognosis in TNBC patients [125]. Moreover, the scavenging of NOX1-related BLT2-mediated ROS by NAC treatment (5 mM) was shown to effectively induce cell apoptosis in the MDA-MB-468 TNBC cell line [123]. 

In addition, down-regulation of the ROS scavenger *methionine sulfoxide reductase A* (*MsrA*), has been associated with advanced cancer in human breast cancer tissues, and the same study indicated that increased ROS production by *MsrA* knockout in a TNBC cell line (MDA-MB-231) led to a more aggressive phenotype in both 3D-culture and tumor xenograft models [126]. Both the mitochondria and the NOX system were involved in ROS hyperproduction due to loss of *MsrA*, which led to decreased PTEN expression, activation of the phosphoinositide-3-kinase pathway, and increased vascular endothelial growth factor (VEGF) production [126]. 

High NOX4 expression has also been shown to increase mitochondria-associated ROS levels, thus resulting in the stabilization of *matrix metalloproteinase-9 (MMP-9)* mRNA [124]. This high MMP-9 expression significantly contributed to cancer metastasis and was linked with invasive properties of MDA-MB-231 cells [124]. In addition, cell adhesion and migration are largely dependent on the binding of integrin to extracellular matrices [127]. High β1 integrin expression has been correlated with low survival rates and advanced metastatic status in TNBC [128]. Studies have implicated the involvement of NOX complexes in integrin-mediated ROS production [127,129]. For instance, the binding of integrin α2β1 to type IV collagen in a human adenocarcinoma cell line was shown to result in the activation of NOX1 [130]. Integrin engagement activates ROS production via NOX1, which causes cancer cells including MDA-MD-231 cells to escape anoikis and survive [131]. Moreover, enhanced adhesion of MDA-MB-231 cells to proteins of extracellular matrices after radiation therapy has been observed and correlated with high ROS production and surface expression of active β1 integrin [132]. The same study demonstrated that treatment with NAC (10 mM) could inhibit this radiation therapy-induced cell adhesion. These results demonstrate that gene expression changes leading to high NOX-related ROS generation increase cell survival and induce invasive and metastatic phenotypes in TNBC, and that the reduction of ROS via NAC treatment can suppress the invasive and migratory behavior of TNBC cells. 

#### 5.1.3. High ROS Production by Cancer Stem-Like Cells in TNBC

Tumors contain phenotypically and functionally heterogeneous cancer cell populations. Consequently, the development of drug resistance and a metastatic phenotype in TNBC may be related to the presence of rare type of cancer cell referred to as cancer stem-like cells (CSCs) or tumor-initiating cells. These produce high amounts of ROS via mitochondrial OXPHOS and demonstrate self-renewal capability and aggressive characteristics such as high levels of metastasis, a tendency towards relapse after treatment, and resistance to chemotherapy [133,134,135]. 

Following chemotherapy, *c-MYC* and *induced myeloid leukemia cell differentiation protein* (*MCL1*) are frequently found to be co-overexpressed in chemotherapy-resistant human TNBC tissues as well as CSCs derived from TNBC cell lines (MDA-MB-436 and SUM159PT) [133]. Both c-MYC and MCL1 have been found to additively increase mitochondrial OXPHOS and to dramatically elevate ROS production, thus contributing to the maintenance of CSCs and the stabilization of hypoxia-inducible factor-1α (HIF-1α) [133] (Section 5.2.3). During chemotherapy, TNBC cells activate OXPHOS to induce hypoxia pathways associated with drug resistance; thus, scavenging ROS may decrease ROS overload and prevent the development of the hypoxic phenotype as well as drug resistance. The dependence of CSC maintenance upon ROS is further demonstrated by the effective treatment of CSCs within TNBC cell lines using a re-engineered CAT [136]. 

In addition, c-MYC has been shown to drive the dysregulation of fatty acid β-oxidation (FAO), and TNBC cells that over-express c-MYC have been shown to exhibit bioenergetic reliance upon FAO [137]. Functional mitochondria are crucial for the maintenance of stemness, and CSCs also rely on mitochondrial OXPHOS for their survival in other cancer types, including liver and head and neck cancer [138,139]. Furthermore, elevated FAO-catalyzed mitochondrial ROS production in CSCs has been reported to promote cancer metastasis [135]. Therefore, drug treatment can induce the acquisition of CSC properties in TNBC and, thus, contribute to metabolic changes, ROS-dependence in stemness, and drug resistance. This, in turn, suggests that NAC treatment can effectively target the stem cell subpopulation in TNBC. 

### 5.2. The Interplay between Cancer Cells and the Tumor Microenvironment via ROS

**Figure 2:**
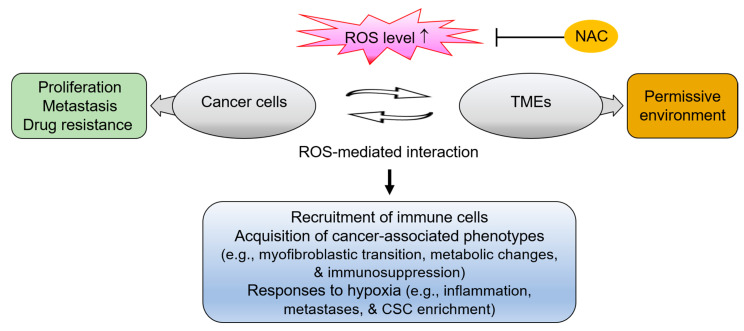
The involvement of reactive oxygen species (ROS) in the interplay between cancer cells and the tumor microenvironment (TME). The ROS-triggered signaling actively involves in interaction between cancer cell and the TME in TNBC. These ROS-mediated interactions induce the recruitment of immune cells, conversion of stromal cells into cancer-associated phenotypes (e.g., myofibroblastic transition, metabolic changes, and immunosuppression), and hypoxic responses (e.g., metastases, inflammation, and cancer stem-like cell (CSC) enrichment), thereby creating permissive TMEs and promoting malignant progression of cancer. N-acetylcysteine (NAC) treatment can effectively interfere with cancer cell-TME interactions by suppressing the ROS signaling that mediates the invasive, drug-resistant, metastatic properties of TNBC cells.

A high ROS content in cancer cells can be transferred to the surrounding TME in a constant interaction that greatly influences cancer progression. For example, *BRCA1* mutations induce oxidative stress in the TME [140], and a high ROS content can arise from high expression and/or activation of NOXs, or from a deficiency of antioxidant enzymes such as GPX, in the stromal cells [141,142]. Regardless of the source, high levels of ROS actively contribute to interaction between cancer cells and the TME, thus leading to more permissive microenvironment toward highly proliferative, metastatic, and drug-resistant properties of cancer (Figure 2).

#### 5.2.1. Conversion to the Cancer-Associated Fibroblast Phenotype via ROS 

Cancer-associated fibroblasts (CAFs) are a main component of the TME and actively contribute to cancer growth and malignancy by secreting various growth factors and chemokines [87,143,144,145]. The CAFs are known to exhibit a myofibroblast phenotype, and the abundance of stromal myofibroblasts that express α-smooth muscle actin (α-SMA) has been shown to predict poorer overall survival rates in various types of cancer, including breast cancer [146,147,148,149]. Although the origin of CAFs remains controversial, they are considered to be largely derived from the activation of resident fibroblasts [145]. In this respect, ROS play fundamental roles in the activation and conversion of fibroblasts into CAFs [141,146,150,151]. Specifically, ROS have been shown to promote the conversion of fibroblasts into myofibroblasts through the up-regulation of HIF-1α and CXCL12, an effect that was reduced by long-term NAC treatment (0.5 mM for 20 days) [151]. In addition, the conversion of fibroblasts into fibrotic myofibroblasts via NOX-4-dependent ROS production in association with transforming growth factor TGF-β1 (TGF-β1) has been suppressed by treatment with NAC (5 mM), either before or after treatment with TGF-β1 [141,146,150,152]. 

In addition to inducing myofibroblast transition, the large amounts of ROS produced by cancer cells induces metabolic changes in the neighboring fibroblasts [153,154]. In an in vitro co-culture model, ROS derived from cancer cells (MCF-7 cells) induced autophagy in fibroblasts and consequently reduced the expression of Caveolin-1 (Cav-1), which led to the development of myofibroblast characteristics (i.e., upregulation of *α-SMA*, *calponin*, and *vimentin*) [153,154,155]. Under these co-culture conditions, Cav-1 in fibroblasts was targeted to the lysosome for its autophagic degradation in response to high ROS levels, which could be prevented by treatment with NAC or with an autophagy inhibitor (chloroquine) [92,155]. The metabolic switch between cancer cells and fibroblasts also involves ROS. For instance, H_2_O_2_ secreted by cancer cells causes oxidative stress in adjacent fibroblasts, which leads to decreased mitochondrial activity and increased glucose uptake, thus creating a dependence on aerobic glycolysis in fibroblasts [153]. In contrast, an enhancement in mitochondrial activity has been observed in cancer cells (e.g., MDA-MB-231 and MCF7 cells) during co-culture [153]. Similarly, in a xenograft mouse model where MDA-MB-231 breast cancer cells were co-injected with wild-type or Cav-1-deficient fibroblasts, the Cav-1-deficient fibroblasts led to enhanced glycolytic enzyme synthesis to provide energy-rich metabolites (e.g., lactate), thus increasing cancer growth and angiogenesis [156]. Hence, Cav1-deficient CAFs, induced by high ROS levels, exhibit a shift towards aerobic glycolysis. 

Notably, a loss of stromal Cav-1 expression is a strong predictor of poor clinical outcome in TNBC and basal-like breast cancers [157]. The *BRCA1* mutation in breast cancer cells has been shown to significantly increase ROS levels in adjacent fibroblasts, thus leading to decreased Cav-1 expression and increased expression of monocarboxylate transporter 4 (MCT4), which is the main exporter of L-lactate from cells [140]. These changes were reversed by both wild-type *BRCA1* and NAC treatment. Furthermore, Cav-1 has been shown to be a negative regulator of NOX proteins, such that reduced Cav-1 expression leads to increased NOX expression and, hence, a further increase in ROS production [158]. Taken together, the results described in this section indicate that the large quantities of ROS produced by cancer cells lead to myofibroblastic transition and a dependence on aerobic glycolysis in neighboring stromal fibroblasts, thus providing an energy-rich cancer-promoting microenvironment. 

#### 5.2.2. Conversion to Tumor-associated Macrophage Phenotype via ROS 

In addition to CAFs, breast TMEs include tumor-associated macrophages (TAMs). These are a critical component of the TME and greatly influence cancer progression and the therapeutic response [159]. High ROS levels derived from cancer cells contribute to the recruitment of macrophages into the TME and to their conversion to a more permissive macrophage phenotype [91,105]. For instance, ROS were shown to increase the activation of aryl hydrocarbon receptor (AhR), thus leading to increased transcription of antioxidant enzymes and epidermal growth factor receptor (EGFR) ligand (amphiregulin) in malignant mammary cells [91]. In this study, AhR and amphiregulin were shown to regulate the production of chemokines (e.g., granulocyte colony-stimulating factor, CXCL1, CXCL2, and C-C motif chemokine ligand 5) to attract monocytes into the TME in a *BRCA1*-deleted mouse mammary tumor model. Notably, the expression of these chemokines and the infiltration of monocyte lineage cells were also correlated with the ROS levels in *BRCA1* mutation-associated human breast cancer tissues in the same study. These results are further supported by those of another study in which continuous treatment with an ROS inhibitor (butylated hydroxyanisole, BHA) efficiently blocked the recruitment of monocytes into the TME and improved the prognostic values in various transgenic mouse models, including *MMTV-PyMT*-induced breast cancer models [93]. 

Cancer immune escape refers to the avoidance of immune control of cancer growth and spread and is considered an important strategy for cancer survival and development [160]. The programmed death ligand-1 (PD-L1) signaling pathway is an important component of cancer immune escape [160,161]. Antioxidant depletion or the generation of ROS by treatment with BSO or paclitaxel has been shown to positively regulate mRNA and protein levels of PD-L1 in both human and mouse macrophages in vitro [105]. These PD-L1-expressing macrophages have immunosuppressive and angiogenic properties due to the production of immunosuppressive cytokines (e.g., interleukin (IL)-4, IL-10, and IL-17) and VEGF, respectively [105]. In this study, the administration of paclitaxel for the treatment of the spontaneous-TNBC analogue *BRCA1*/*p53*-deleted mouse model mammary tumors induced PD-L1 expression in the TAMs, thus leading to an immunosuppressive TME. NF-κB signaling in response to ROS accumulation mediated the promotion of PD-L1 transcription and the release of immunosuppressive chemokines. In another study, NOX-mediated ROS generation was shown to play a critical role in macrophage differentiation into immunosuppressive M2 macrophages, whereas inhibition of ROS by BHA treatment specifically blocked the differentiation into M2, but not M1 macrophages [93]. Thus, ROS act as paracrine signaling molecules to recruit TAMs into the TME and thus alter the TAM phenotype to exhibit more immunosuppressive characteristics.

#### 5.2.3. Activation of Hypoxic Responses via ROS-Mediated Tumor-Stromal Interaction

A hypoxic microenvironment surrounding cancer cells can also promote invasion, metastasis, and resistance to therapy [162,163,164]. Hypoxia-inducible factors (HIFs) are critical transcription regulators that respond to hypoxia [165]. These include the oxygen-regulated HIF-1α, HIF-2α, and HIF-3α subunits, and the constitutively expressed HIF-1β subunit [164]. The HIFs are subject to prolyl hydroxylation, ubiquitination, and proteasomal degradation, all of which are inhibited under hypoxic conditions; hence, the latter conditions facilitate the stabilization and accumulation of HIF-α protein [164,165]. High expression of HIFs or HIF-regulated genes is particularly common in basal-like TNBCs, thus suggesting that hypoxia may play a relevant role in promoting aggressive behaviors of these tumors [166,167]. In fact, the activation of HIF has been suggested as a mechanism by which TNBCs can acquire invasiveness and metastatic propensity [168,169,170]. Likewise, HIFs are required for the enrichment of chemotherapy-resistant CSCs in TNBC [163] (Section 5.1.3). 

Hypoxic conditions also mediate the tumor-stromal interactions that lead to the elevation of cytokines, in turn, promote metastatic phenotypes in TNBC [171,172]. For instance, hypoxia has been shown to induce the recruitment of macrophages and mesenchymal stem cells (MSCs) by the production of macrophage colony-stimulating factor 1 via the interaction between TNBC cells and MSCs [171]. Hypoxia is also involved in the interaction between TNBC cells and breast CAFs via interleukin-1β (IL-1β) and interleukin receptor 1 type 1 to induce metastatic gene expression and invasive properties in TNBC cells [172]. In particular, Il-1β is highly induced in hypoxia [172] and is one of major highly-elevated proteins in TNBCs [173]. Hence, tumor-stromal inflammation induced by hypoxia stimulate the pro-metastatic phenotypes in TNBC [173].

Notably, ROS are increased in response to hypoxia and inhibit the ability of prolyl hydroxylases to hydroxylate HIFs [174]. Therefore, ROS production involving the mitochondria is required for the stabilization of HIF-1α and the subsequent transduction of the hypoxia-mediated signaling [165,175]. Hypoxia has been shown to induce ROS production in MDA-MB-468 cells, and treatment with NAC (10 mM) was shown to reverse hypoxia-induced up-regulation of N-cadherin and plasminogen activator inhibitor-1, the activation of EGFR, and cell motility [104]. In addition, exposure of MDA-MB-231 cells to hypoxia led to increased levels of ROS, the activation of two main HIF-1α transduction regulators (extracellular signal-regulated kinases and AKT), and upregulation of c-Fos protein, whereas treatment with NAC (0.3 mM) prevented these changes induced under the hypoxic conditions [172]. Furthermore, treatment with drugs that differ in their mechanisms (e.g., paclitaxel and gemcitabine) has been shown to induce hypoxic responses and CSC enrichment in TNBC cell lines (MBA-MB-231, SUM-149, and SUM-159), and this was mediated by increased ROS levels [176]. Therefore, ROS mediated hypoxic response in TNBC, and NAC could effectively inhibit the transduction of hypoxia-mediated signaling. 

As noted above, ROS are actively involved in the interplay between cancer cells and the TME for cancer progression in TNBC, and NAC can suppress this process by interfering with the ROS-mediated cancer cell-TME interaction. A pilot study has demonstrated that short-term treatment (14-27 days) with NAC significantly reduces the level of stromal MCT4 expression and proliferation in breast cancer patients [124]. Therefore, NAC has been shown to be effective in the metabolic modulation of breast TME at the clinical level. 

Traditional or standard chemotherapy is currently the main systemic treatment option for TNBC. Although optimal regimens still need to be established, chemotherapy regimens based on anthracyclines (e.g., doxorubicin) and taxanes (e.g., paclitaxel) represent the mainstay of TNBC treatment [177]. However, although TNBC is initially sensitive to chemotherapy, progressive resistance is a common problem that leads to disease recurrence and a poor outcome, thus limiting the benefits of standard chemotherapy [178]. The inclusion of NAC in standard chemotherapy can potentially provide additional benefits for TNBC patients, although it should be ensured that the NAC treatment does not interfere with the effectiveness of the chemotherapy. Currently, the combinational use of NAC is more focused on its effects in terms of preventing or relieving the adverse side effects of standard chemotherapy, such as peripheral neuropathy due to taxane chemotherapy [179] or cardiotoxicity caused by anthracycline chemotherapy [180,181]. Although some preclinical studies on the combination of NAC and standard chemotherapy have been published, the reported effects are inconsistent. For instance, pretreatment with NAC has been reported to potentiate the doxorubicin-induced phosphorylation of p53 and ATM, thus increasing their inhibitory effects upon cell proliferation and migration in ovarian cancer cells [182]. In addition, NAC has been shown to prevent NF-κB activation by gemcitabine and to improve the efficacy of gemcitabine in pancreatic cancer cells that were implanted in athymic nude mice [183]. By contrast, NAC has been shown to negatively alter the chemotherapeutic effectiveness of paclitaxel in a lung cancer cell line by decreasing the ROS levels, thus preventing paclitaxel-induced apoptosis [184]. However, it should be noted that studies using cell culture models may be misrepresented due to the absence of in vivo-like TMEs, which are an important component of the benefits of NAC. The potential interference of NAC with the effectiveness of standard chemotherapy critically depends on whether the specific chemotherapy depends on ROS production. For example, the anticancer efficacy of radiotherapy does not depend on ROS and, hence, NAC treatment reduces the levels of radiotherapy-induced ROS but does not interfere with radiotherapy-induced cell death [185]. In fact, high ROS is often associated with resistance to standard chemotherapy probably due to their involvement in CSC enrichment and permissive TME formation [91,163,186] (Section 5.1.3 and Section 5.2). Moreover, NAC treatment can be particularly effective in the inhibition of the tumor-stromal interaction and the reduction in CSC population, thereby decreasing chemotherapy-induced resistance and aggressiveness in TNBC. 

## 6. Conclusions 

NAC is a membrane-permeable antioxidant and has been safely used as a mucolytic and antidote for acetaminophen. However, its use in cancer treatment has resulted in varying outcomes ranging from beneficial to detrimental largely because ROS can mediate both cancer-promoting and cancer-suppressing signaling. In this respect, particularly high ROS levels are generated by TNBC cells due to gene mutation/inactivation (e.g., *BRCA1* and *TP53*), gene expression changes, and CSC enrichment. This ROS accumulation mediates the cellular signaling necessary to maintain the survival and promote their metastatic capacity and drug resistance. Furthermore, high ROS levels derived from TNBC cells can mediate interaction between cancer cell and the TME to induce the formation of permissive TME and hypoxic responses that linked to their aggressive behavior. However, treatment with NAC effectively suppresses the ROS production and ROS-mediated signaling that contribute to cell survival, metastasis, and drug resistance, thereby inhibiting cancer progression. Meanwhile, TNBCs exhibit aggressive phenotypes, and standard chemotherapy remains the mainstay of TNBC treatment that often generate resistance and relapse. Therefore, the additional use of NAC can be an effective strategy for treating those TNBCs. However, further studies are required in order to define better molecular signatures for the identification of specific subset of TNBC patients who can benefit the most from NAC therapy and to monitor its efficacy. Moreover, for the use of NAC to be clinically relevant, further studies need to examine the potential interference of NAC with the effectiveness of standard chemotherapy, the most suitable time for NAC administration relative to the standard chemotherapy, and the appropriate treatment duration.
